# An Observational Study Comparing the Effects of Chloroquine and Artemisinin-Based Combination Therapy on Hematological Recovery in Patients With Plasmodium vivax Malaria

**DOI:** 10.7759/cureus.47127

**Published:** 2023-10-16

**Authors:** Sharada Shri Rao, Kuladeepa Ananda Vaidya, Aashish K Sharma

**Affiliations:** 1 Pharmacology, Srinivas Institute of Medical Sciences and Research Centre, Mangalore, IND; 2 Pathology, Srinivas Institute of Medical Sciences and Research Centre, Mangalore, IND

**Keywords:** haematological profile, artemisinin based therapy, chloroquine, plasmodium vivax, malaria treatment

## Abstract

Introduction

Malaria, a common parasitic disease in tropical regions, produces hematological changes in patients. In the present study, we compare hematologic recovery between the chloroquine and artemether-lumefantrine treatment groups of patients with *Plasmodium vivax* malaria.

Methodology

This was a cross-sectional observational study comparing hematological parameters, including total and differential white blood cell counts, and platelet counts between two patient groups: one group with 48 patients receiving chloroquine, and the other with 47 patients receiving the artemether-lumefantrine combination. Both groups received primaquine to combat the hypnozoite stage.

Results

The rate of platelet count recovery was significantly faster in patients treated with the artemether-lumefantrine combination (p-value 0.002). Rates of recovery of the total white blood cell count and neutrophil count were faster with the artemether-lumefantrine combination, while the recovery of the lymphocyte count was faster in patients treated with chloroquine. However, these changes were statistically insignificant (p-values = 0.69, 0.42, and 0.65, respectively).

Conclusion

Based on hematological recovery, artemisinin combination therapy may be preferred over treatment with chloroquine in cases of *P. vivax* malaria. However, factors such as the adverse effect profile, cost-effectiveness, and chloroquine resistance need to be considered for the practical applicability of the same.

## Introduction

Malaria is one of the common parasitic diseases affecting humans, caused by various species of *Plasmodium*, namely *P. falciparum*, *P. vivax*, *P. ovale*, *P. malariae*, and *P. knowlesi*. Malaria is prevalent in tropical regions. India harbors both *P. vivax *and *P. falciparum*, contributing to 90% of malarial cases in the South East Asian region [1]. Hematological changes are among the most common complications in malaria and play a significant role in malaria pathogenesis. These changes involve major cell types such as erythrocytes, leukocytes, and thrombocytes [2]. Chloroquine has been the drug of choice for both the treatment and chemoprophylaxis of malaria, although *P. falciparum* has developed drug resistance. The treatment for radical cure of *P. vivax *malaria includes chloroquine (to eradicate erythrocytic forms) and primaquine (to eradicate hypnozoites) [3]. According to WHO guidelines, the combination of chloroquine and primaquine is considered the standard treatment for *P. vivax* malaria. 8-aminoquinoline (e.g., primaquine) is the only drug with significant activity against hypnozoites [4]. Several artemisinin-based combination therapies (ACT) have shown high efficacy against both chloroquine-sensitive and resistant *P. vivax* [5].

Changes in blood cell parameters are well-known features of malarial infestation. These can manifest as anemia, thrombocytopenia, and leukocytosis or leukopenia [6]. In a study by Ngole et al., in which patients treated with sulphadoxine and pyrimethamine were compared with patients treated with artesunate and amodiaquine, patients with *P. falciparum* infestation treated with the latter showed better hematological recovery [7]. A study conducted by Quique Bassat has shown rapid clearance of *P. vivax* parasitemia and fever with the artemether-lumefantrine combination compared to chloroquine. The study also recommends its use in areas with chloroquine-resistant *P. vivax* malaria, especially in places where parasitological differentiation is not routinely performed or where only clinical diagnosis is used [8].

In this study, we compare the effects of chloroquine against the artemether-lumefantrine combination on hematological recovery in patients with *P. vivax* malaria.

## Materials and methods

This was an observational study conducted at Srinivas Institute of Medical Sciences and Research Centre, a tertiary healthcare hospital in Dakshina Kannada district, Karnataka state, India. The study was initiated after obtaining written approval from the Institutional Ethics Committee. Only those patients satisfying the inclusion and exclusion criteria were selected for the study. The study was carried out among patients with *P. vivax *malaria confirmed by standard blood tests, either by examination of a peripheral smear stained with Leishman stain or by detecting the parasite by fluorescent microscopy.

The patients were divided into two groups: one group consisted of patients with *P. vivax *malaria treated with chloroquine tablets (600 mg at the time of diagnosis, followed by 600 mg on the next day, and 300 mg on the third day) and tablet primaquine (15 mg once daily from the day of diagnosis up to 14 days). The other group consisted of patients with *P. vivax *malaria treated with a fixed-dose combination tablet of artemether and lumefantrine (four tablets twice daily for three days, each tablet with 20 mg of artemether and 120 mg of lumefantrine), and primaquine (15 mg once daily from the day of diagnosis up to 14 days). Primaquine course had to be given in both groups to target the hypnozoite stage to prevent relapse later on. Fifty patients were included in each group.

A proforma containing patient demographic data (age, gender, medical history) and drug data (generic/brand name, dosage form, dose frequency, and route of administration) was utilized. Clinical and laboratory data of each patient recorded before the treatment, on days seven and fourteen, were included.

Values of the total white blood corpuscle count (WBC), differential WBC count, and platelet count were obtained by running EDTA anticoagulated whole blood samples of the subjects in the 5-part fully automatic Coulter counter-Horiba Yumizen H500 machine. Each of the results obtained was manually verified using Leishman stained peripheral smear by the reporting Pathologist. The data thus obtained were tabulated for statistical analysis.

Scope of the study

This study helps compare the effects of chloroquine and ACT on hematological parameters in patients with *P. vivax* malaria.

Inclusion criteria

Patients of ages greater than 15 years, including both genders diagnosed with *P. vivax* malaria confirmed by peripheral smear examination/malaria parasite fluorescent test, treated with either chloroquine or artemether-lumefantrine combination were included in the study.

Exclusion criteria

Patients who had taken anti-malaria treatment or had a malaria infection within the previous 12 months, patients in whom there was another possible cause for their fever or suffered from some chronic disease, and patients with non-*P*.* vivax *malaria were excluded.

Statistical analysis

Data analysis was carried out using IBM SPSS Statistics for Windows, Version 18 (Released 2009; IBM Corp., Armonk, New York). Patients with hematological recovery in both groups were compared using the Chi-square test. A p-value of less than 0.05 was considered statistically significant.

## Results

A total of 100 patients satisfying the selection criteria were included in the study, with 50 patients in each group. Follow-up investigations were not available for three patients in the artemether-lumefantrine group and two patients in the chloroquine group. The demographic details of each group are presented in Table 1.

**Table 1:** Comparison of demographics in the two treatment groups.

Characteristics	Chloroquine, n (%)	Artemether-lumefantrine, n (%)
Number of patients included in the study	50 (100%)	50 (100%)
Number of patients who completed the study	48 (96%)	47 (94%)
Number of patients who belonged to the male gender	32 (64%)	29 (58%)
Number of patients who belonged to the female gender	18 (36%)	21 (42%)
Mean age (years)	43.31±13.43	40.62±12.08

The mean age of the patients in both groups was 43.31±13.43 and 40.62±12.08 years, respectively. Age in both groups was comparable (p-value = 0.29).

The baseline parameters of each patient were recorded, and the patients were categorized as shown in Table 2.

**Table 2:** Patients were categorized based on total WBC count, platelet count, and absolute counts of neutrophils and lymphocytes under the two groups before the treatment.

Baseline parameters (number of patients)		Chloroquine, n=48 (100)%	Artemether-lumefantrine, n=47 (100%)
WBC	Normal	22 (45.83%)	15 (31.91%)
	Low	22(45.83%)	25 (53.20%)
	High	4 (8.34%)	7 (14.89%)
Neutrophil count	Normal	13 (27.08%)	15 (31.91%)
	Low	29 (60.42%)	27 (57.45%)
	High	6 (12.50%)	5 (10.64%)
Lymphocyte count	Normal	25 (52.08%)	22 (46.81%)
	Low	13 (27.08%)	17 (36.17%)
	High	10 (20.84%)	8 (17.02%)
Platelet count	Mild thrombocytopenia	25 (52.08%)	21 (44.68%)
	Moderate thrombocytopenia	11 (22.92%)	14 (29.79%)
	Severe thrombocytopenia	6 (12.50%)	7 (14.89%)
	Normal	6 (12.50%)	5 (10.64%)

**Figure 1:**
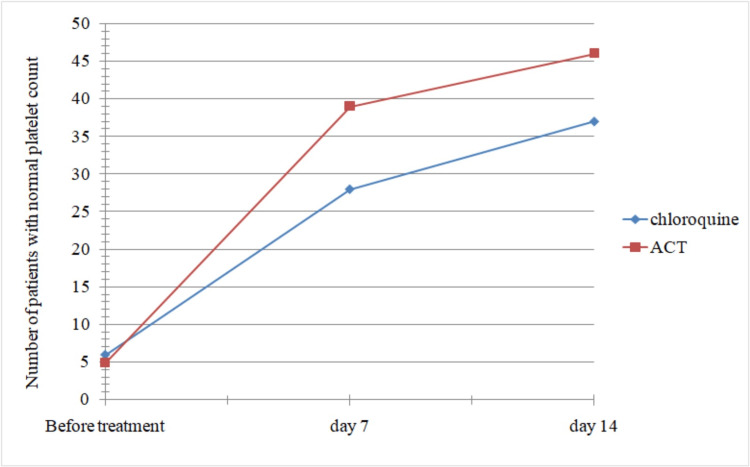
Comparison of the two groups of patients with reference to time taken for return to normal platelet count. ACT: artemisinin-based combination therapy.

Patients on ACT showed a significantly faster rate of improvement in platelet count compared to the chloroquine group at the end of the first and second week (p-value = 0.002), as evident from Figure 1.

**Figure 2:**
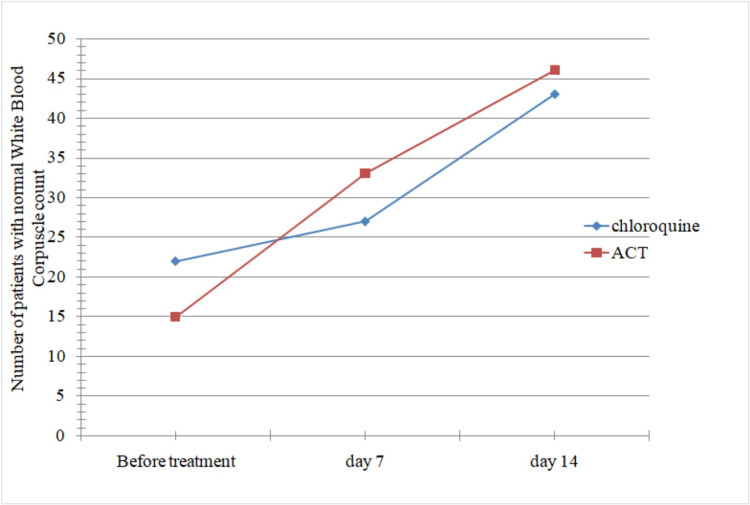
Comparison of two groups of patients with reference to time taken for return to normal WBC count. ACT: artemisinin-based combination therapy.

By the end of day 7 and day 14, more patients on ACT therapy showed normal WBC count compared to those on chloroquine therapy. Hence, response to therapy is faster in the earlier group compared to the latter, as seen in Figure 2, though the difference was not significant (p-value = 0.69).

**Figure 3:**
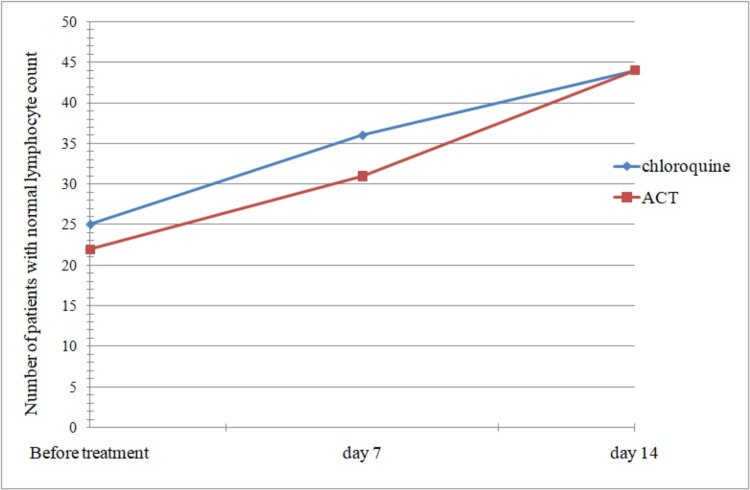
Comparison of the two groups of patients with reference to time taken for return to normal lymphocyte count. ACT: artemisinin-based combination therapy.

By day 7, more patients on chloroquine-based therapy showed a normal lymphocyte count compared to those on ACT, though by the end of 14 days, both groups had an equal number of patients with normal lymphocyte count, as seen in Figure 3. However, this was not statistically significant (p-value = 0.65).

**Figure 4:**
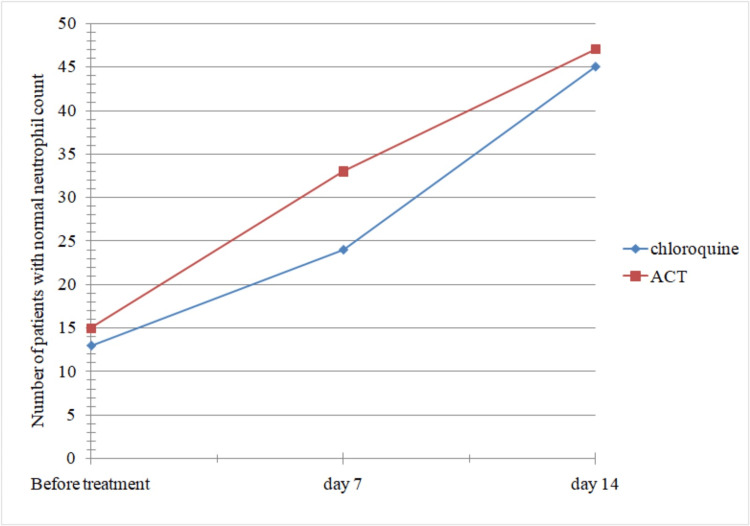
Comparison of the two groups of patients with reference to time taken for return to normal neutrophil count. ACT: artemisinin-based combination therapy.

By day 7 and day 14, more patients on ACT showed normal neutrophil count compared to those on chloroquine therapy, as seen in Figure 4. Hence, the response to therapy is faster in the earlier group compared to the latter. However, the difference was statistically insignificant (p-value = 0.42).

## Discussion

The difference in the number of patients with normal platelet counts between both groups at the end of the first and second weeks is significant (p < 0.05). A study conducted by Martinez-Salazer et al. [9] in 2014 included 311 patients with *P. vivax* infection. They found that patients with severe thrombocytopenia exhibited higher parasitemia than those without thrombocytopenia. They documented that alterations in liver function tests correlated with changes in platelet count and indices. They also found that thrombocytopenia accompanies malarial complications, especially hepatic dysfunction. This underscores the importance of faster platelet recovery, which our study found to be more pronounced in the artemether-lumefantrine group compared to the chloroquine group. This suggests that ACT is a preferable choice for hastening platelet recovery and reducing the likelihood of patients requiring platelet transfusion.

While a greater number of patients in the artemether-lumefantrine group had normal white blood cell (WBC) and neutrophil counts compared to the chloroquine group, the rate of recovery following treatment was not significantly different between the groups (p > 0.05). Although lymphocyte recovery was found to be faster in the chloroquine group, it was not statistically significant (p > 0.05).

Hematological recovery was faster in patients on ACT compared to those on chloroquine. This aligns with a study conducted by Ngole et al where patients on ACT exhibited the highest hematological recovery [7].

The study by Nicholas et al. shows a shorter time to parasite clearance in patients receiving ACT compared to those on chloroquine [5]. In our study, although parasite clearance time was not studied, faster platelet and WBC recovery in the ACT group indirectly reflects the same.

A study by Douglas et al [5] demonstrates that artemisinin derivatives are highly effective against *P. vivax *and may offer advantages over chloroquine for this species. It also emphasizes that continued use of chloroquine rather than ACTs for the treatment of *P. vivax *malaria can lead to the emergence and spread of chloroquine resistance. It proposes a unified ACT-based strategy for both species in all co-endemic settings where there is a high frequency of misdiagnosis and an increase in chloroquine-resistant *P. vivax*.

A study conducted by Pukrittayakamee et al. [10] indicates that artesunate and artemether have significantly higher *P. vivax* parasite reduction ratios than chloroquine, further supporting our study.

## Conclusions

Based on hematological recovery (particularly that of platelets), artemether-lumefantrine combination therapy can be preferred over chloroquine in the treatment of *P. vivax* malaria where there are cases of chloroquine resistance and where mixed *P. falciparum* and *P. vivax* infection are common. However, the adverse effect profile and cost-effectiveness of drugs need to be studied to emphasize the same. As the present study included a small number of patients within a limited period of time, further studies with a large number of patients have to be carried out to establish the data.
